# My Student

**Published:** 1887-10

**Authors:** J. D. Moody

**Affiliations:** Mendota, Ill.


### MY STUDENT.

BY J. D. MOODY, D. D. S., MENDOTA, ILL.

I have been chary of students. Have had applicants plenty, but I am determined not to induct a man into the profession whom I believe to be unfit in any particular, or one of whom I cannot expect the very best. There are enough mediocre men already. I was proud, years ago, to receive the first applicant. It was something to have a student. But I soon repented my action, and since that time have uniformly said nay to all comers. I always did it pleasantly, however, and gave my reasons for it, and as for qualifications, I placed them at so high a mark as to discourage any but the greatest enthusiast.

I have felt that I was in some sense a door-keeper to the profession, and remembering my own easy entrance and the dire consequences of that same "easy entrance," I have determined to admit no one who cannot show qualifications entitling him to admission. The time was when the practitioner was chargeable with the influx of so much imperfect material, but things are different now. Studentship is largely relegated to the colleges, and these, in their struggle for existence, do not scan closely enough those who are passing through their doors. It is true that we have better material and better preparation than ever before, and yet the scrutiny is not close enough. Many unfit persons are still coming in. Unfit from a lack of knowledge of foundation principles, unfit by reason of mental organization, unfit from lack of moral principle.

But although we do not have as many students as formerly, we can exercise a great influence, both by our personal example and by our advice to those desiring to study, and as well by positive refusal to all but those properly endowed. I do not know but it is our duty to advise with the college to which a young man intends applying for entrance. We know the young man who goes from our town, better than the college secretary who takes his matriculation fee possibly can by a half hour's examination. Then, too, I believe a "good character" should mean something. We can make it mean something. Let me illustrate my meaning by quoting to you "My Student."

A young man came to me wishing to study dentistry. He was a country-bred boy, of good morals and a fair common-school education, rather backward in manner and appearance. To me he did not seem to possess the first qualification for a dentist. I tried pleasantly to discourage him, but he returned to the matter again and again. Finally, thinking his persistence deserved some attention, I enquired of him what he could do. Only one gleam of light appeared—he was handy with tools. I told him he was totally unprepared to study dentistry. Yet I have no doubt he could have entered almost any dental college, and been graduated in two years a mediocre dentist, and probably always have remained such. I told him, however, if he would pursue a course of preparatory study, such as I would lay out for him, and complete it creditably, that I would then lend him what assistance I could. I felt that if after doing the work designated he would still insist on studying dentistry, there must be the right kind of stuff in him. He consented to the conditions, and as soon as I could find the proper place for him, began his school life. I sought for a college where he could have the privilege of choosing his own studies, regardless of any regular course, and at the same time have good facilities for pursuing just those studies which I desired he should have. I was fortunate in finding just the place for him.

I gave him Botany, Zoology, Physics, Chemistry, Microscopy, and Animal and Vegetable Physiology as the required studies. He was to put in two years, of ten months each, on this work. He has just completed the course, and with flattering commendations from the faculty. In connection with physiology, he learned all the bones of the body and something of the arterial and nervous systems, and quite a good deal of hygiene. He received more of chemistry than is usual in dental colleges. In zoology he got a fair knowledge of the lower orders, which, in connection with vegetable physiology, gave him a taste for biological knowledge and a basis upon which he can in the future work out these problems by himself. I kept track of his study, and directed it from time to time as I thought best, consulting, of course, with his preceptors in the matter. He has come back to me prepared to begin, and full of enthusiasm for scientific work, and more than ever determined to be a dentist. There is but one course to pursue. The door should be opened to all such, *and only to such.* He is now fitted to go into college and understand what the lecturers are talking about. He is in position to make the very most of the scientific suggestions thrown out by his teachers. In this I am not urging the cultivating of the intellect to the exclusion of the manual training. He has laid the intellectual foundation, and it now will not require so much of his time at college to learn preliminaries. Consequently, he can devote more attention to the manipulative part of the work, and acquire more strictly dental knowledge than would otherwise be possible in a two years' course. Any spare time that may come to hand can be devoted to perfecting himself in those studies which are not so familiar. Were all students so prepared, it would make easy work for the instructors; it would make grand work for the profession.

In the interim between school days he is in my office learning the names of things, their uses, working in steel, fashioning instruments, moulding in sand, etc., etc.

Possibly a better course of preliminary study could be suggested. Have I asked too much of him—subjected him to unnecessary expense ? Practically, this is a four years' dental course, somewhat akin to the German gymnasium. Speed the day when something like it shall be the law of our land.

